# Recurrent heart failure hospitalizations increase the risk of mortality in heart failure patients with atrial fibrillation and type 2 diabetes mellitus in the United Kingdom: a retrospective analysis of Clinical Practice Research Datalink database

**DOI:** 10.1186/s12872-022-02665-y

**Published:** 2022-05-21

**Authors:** Raquel Lahoz, Ailís Fagan, Martin McSharry, Clare Proudfoot, Stefano Corda, Rachel Studer

**Affiliations:** 1 Novartis Pharma AG, 4056 Basel, Switzerland; 2 Novartis Ireland Limited, 203 Merrion Rd, Dublin, D04 NN12 Ireland; 3 Empower The User, Unit 1B, Trinity Technology & Enterprise Campus, Pearse St, Dublin, D02 KD43 Ireland; 4 Bayer AG, 51368 Leverkusen, Germany

**Keywords:** Heart failure, Atrial fibrillation, Diabetes mellitus, Hospitalization, Mortality

## Abstract

**Background:**

Heart failure (HF) is a global illness and is a leading cause of hospitalizations. Recurrent HF hospitalization (HFH) is associated with increased risk of cardiovascular (CV) and all-cause mortality, thereby burdening the health system. Type 2 diabetes mellitus (T2DM) and atrial fibrillation (AF) are two important comorbidities in patients living with HF. This study aims to assess the association between recurrent HFHs with CV and all-cause mortality in patients living with HF and having AF and/or T2DM.

**Methods:**

This study was conducted using primary care data from the Clinical Practice Research Datalink database with linkage to hospital data and mortality data. Adults living with HF and with at least 1 HFH were identified from January 2010 to December 2014. Patients were grouped based on the number of recurrent HFHs. During follow-up, all-cause mortality or CV mortality for the HF population with AF and T2DM was recorded.

**Results:**

Overall, 32.9% of 2344 T2DM patients and 28.2% of 4585 AF patients had at least 1 recurrent HFH. The patients were relatively elderly and were predominantly male. The mean number of all-cause hospitalizations in HF patients having T2DM and AF, with ≥ 1 recurrent HFH were significantly higher than patients without recurrent HFH. The annualized mortality rates in CV mortality as the primary cause and for all-cause mortality and increased with recurrent HFHs, in T2DM and AF patients. The risk of CV mortality as primary cause and all cause morality were 5.39 and 3.19 times higher in T2DM patients with 3 recurrent HFHs versus no recurrent HFH. Similarly, the risk of CV mortality as primary cause and all cause morality was 5.98 and 4.3 times higher in AF patients with 3 recurrent HFHs versus those with no recurrent HFH.

**Conclusions:**

Recurrent HFHs are strongly associated with CV mortality and all-cause mortality in HF patients with TD2M or AF. The hospitalization rate highlights the need for treatment and disease management, which will improve the course of the disease and help patients stay out of hospital.

**Supplementary Information:**

The online version contains supplementary material available at 10.1186/s12872-022-02665-y.

## Background

Heart failure (HF) is a highly prevalent illness globally, with approximately 64.3million patients in 2017 [1]. A survey conducted in 2018–2019 by the European Society of Cardiology in 42 of its member countries showed that the median incidence of HF was 3.20 cases per 1000 person-years and the median prevalence was 17.2 per 1000 population people. The survey reported that the median number of HF hospitalizations was as high as 2671 per million people annually in these countries [2]. It is estimated that approximately 920,000 people are living with HF in the United Kingdom (UK) [3]. Studies have shown that the incidence and prevalence of HF have increased over the years and with advancing age [4–6]. This increase is attributed to the increasing proportion of elderly in the population with morbid conditions such as hypertension, diabetes, etc., which are also risk factors for the development of HF [7] as well as the improved survival in patients already diagnosed with HF [8]. The economic impact that HF has on the health care system is significant, with the estimated annual cost being approximately $108 billion per annum, of which approximately 60% is due to direct costs, including hospitalization costs [9]. Hospital admission costs are the most expensive cost element in HF and the costs are expected to rise with the increase in prevalence of HF [7, 9]. In the UK, approximately 1%–2% of the National Health Service (NHS) budget is currently spent on HF, with 60%–70% driven by the costs of hospitalization alone [10]. Evidence from published studies has shown that recurrent HF hospitalizations (HFHs) are frequent in patients with HF [11, 12]. An early publication, by the same authors using data from the UK-based Clinical Practice Research Datalink (CPRD) database has shown that recurrent HFH is associated with increased risk of cardiovascular (CV) and all-cause mortality [13], thereby burdening the health system even more. Type 2 diabetes mellitus (T2DM) and atrial fibrillation (AF) are two important comorbidities in patients living with HF. In patients living with HF, the prevalence of T2DM ranges from 10 to 47% and nearly 13%–27% have AF [14, 15]. This study from the CPRD database aims to assess the association between recurrent HFHs with CV and all-cause mortality in patients living with HF and having AF and/or T2DM thus paving a way for holistic management by understanding the disease progression in these HF subpopulations.

## Methods

### Database

Like our study that was published earlier [13], this was a retrospective non-interventional analysis from the CPRD database, conducted using primary care data. The CPRD data was was linked to hospital data and mortality data. The CPRD is a longitudinal primary care database and is the world’s largest database of anonymized primary care medical records, based in the UK [13, 16]. This database contains more than 10 million active patient records drawn from approximately 1100 primary care practices, with data extending as far as 1987 [13, 16]. Furthermore, this database is linked to other electronic health records, such as the Hospital Episode Statistics (HES), practice level Index of Multiple Deprivation (IMD), and the Office of National Statistics (ONS), for the mortality data.

### Study design

**Fig. 1:**
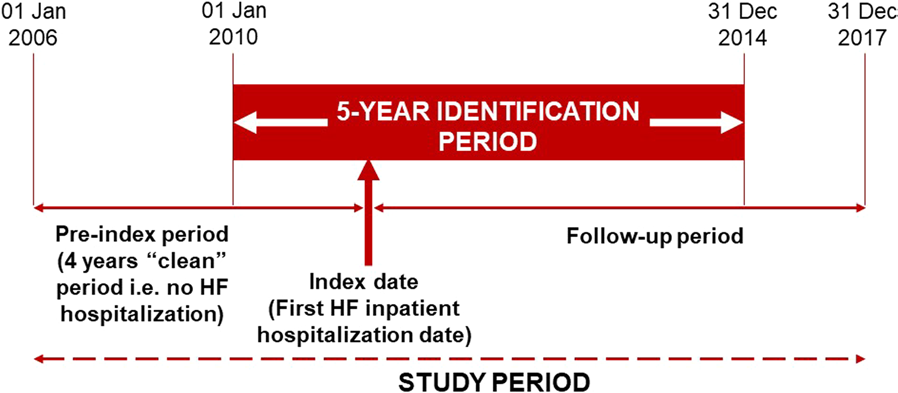
Illustration of the study design. HF, Heart failure

The study design is depicted in Fig. 1. The study period was from January 01, 2006, to December 31, 2017, with the identification period extending from January 01, 2010, to December 31, 2014, during which adult patients living with HF (aged ≥ 18 years) and with at least 1 HFH were identified for inclusion. The index date was defined as the date of first HFH during the identification period. The follow-up period was until the study end (December 31, 2017), death, transfer out date (whichever event was the earliest). For this study, the pre-index period was 4 years prior to the index date; during this time, patients had to have no HFH (“clean” period).

### Eligibility

The patient records were eligible for inclusion based on the following criteria:

- Adult patients with HF (aged ≥ 18 years at index date) that had a diagnosis of AF or T2DM (groups are not mutually exclusive) in the year prior to the index date from either the primary care or HES patient records.
- Patients with records for linkage to the HES, IMD, and ONS death register databases, and with continuous practice registration up-to-standard for at least a year before the index date and the follow-up period (or until death) for each patient.

Patients without acceptable data (as per the quality standards defined by the CPRD database) along with HFH during the 4-year pre-index period were excluded.

For identifying HF diagnoses and HFHs, the researchers used primary care coding schemes (“Read” codes) and the International Classification of Diseases, 10th Revision (ICD-10) codes (I11.0, I13.0, I13.2, I42.0, I42.1, I42.2, I42.9, or I50.X) (Additional file 1).

### Study variables

As mentioned in our previous CPRD publication, patients were grouped based on the number of recurrent HFHs they experienced (0, 1, 2, 3, 4, ≥ 4). A patient who either died after experiencing index HFH during the identification period or who survived until the end of follow-up with no subsequent recurrent HFHs was classified as having 0 or no recurrent HFH [13]. After each recurrent HFH, patients were followed up until death or until the end of study. The group with ≥ 4 HFHs also contained patients with 5, 6, or more HFHs as it had patients who had survived for a relatively long time while experiencing multiple hospitalizations, and it is important to know the different time at risk during event rate calculation.

The baseline characteristics assessed for this cohort, at the index date included age, sex, body mass index (BMI), socioeconomic status etc. (Table 1).

**Table 1:** Demographic and baseline characteristics of patients with HF hospitalization

Characteristics	T2DM			AF		
	Overall	Recurrent HF Hospitalizations		Overall	Recurrent HF Hospitalizations	
		None	≥ 1		None	≥ 1
N	2344	1573	771	4585	3294	1291
Age at index (Mean [SD])	76.4 (10.32)	76.7 (10.4)	75.8 (10.0)	79.6 (10.3)	78.8 (10.3)	79.9 (10.4)
Gender, Male (%)	59.1	58.7	59.9	54.2	55.2	53.9
Any CRT devices prior to index (%)	15.4	14^#^	18.2^#^	16.9	15.3^#^	20.8^#^
Follow-up time (Patient years)	4268.5	2514.2	1753.3	7845.6	5142	2703.6
*Smoking status (%)*						
Current smoker	11.9	12.5	10.5	9.8	9.8	9.8
Never smoker	41.5	41.0	42.4	41.4	41.2	41.9
Former smoker	44.8	44.6	45.1	41.6	41.3	42.2
Missing/unknown	1.9	1.9	1.9	7.3	7.7	6.1
BMI (Mean [SD])*	30.9 (7.0)	30.8 (7.1)	31.1(6.9)	28.5(6.8)	28.3(6.9)	28.9(6.5)
Underweight: < 18.5 kg/m^2^ (%)	0.9	0.9	1.0	1.5	1.6	1.1
Normal weight: > = 18.5 and < 25 kg/m^2^ (%)	14.6	15.1	13.6	16.9	16.8	17.1
Overweight: > = 25 and < 30 kg/m^2^ (%)	23	23	23	17.2	16.6	18.8
Obese: > = 30 kg/m^2^ (%)	40.7	39.7	42.7	20.2	19.1	23.1
Missing/Unknown (%)	20.8	21.4	19.7	44.2	45.8	40.0
Comorbidities (%)*						
AF	49.9	49.1	51.5	100	100	100
Anemia	25.2	24.5	26.6	20.2	19.4^#^	22.2^#^
Angina pectoris	28.7	27.3	31.7	23.4	22.8	25.1
Dyslipidemia	30.5	30.3	30.9	19.8	18.8^#^	22.3^#^
Hypertension	76.5	76.0	77.4	68.0	67.0^#^	70.6^#^
Oedema	23.9	24.9	21.9	23.3	24.0	21.5
Chronic pulmonary disorder	31.5	31.7	31.1	27.5	26.9	28.9
Diabetes	100	100	100	26.2	24.1^#^	31.3^#^
Myocardial infarction	21.2	20.9	21.9	16.4	16.2	16.8
Renal disease	38.1	37.5	39.4	30.1	29.7	31.2
Pneumonia	16.3	16.5	16.1	17.6	18.4^#^	15.7^#^
Ischaemic heart disease	58.2	57.1	60.3	47.3	46.2	50.1
Any concomitant medications at index (%)	85.7	84.8	87.4	82.5	81.3	85.6
ACEi	53.1	53.2	52.9	47.6	46.5	50.4
ARB	23.7	22.5^#^	26.2^#^	19.0	17.9^#^	21.7^#^
ACEi/ARB	74.2	73.1	76.4	65.0	63.1^#^	70.1^#^
Beta-blockers	53.5	52.4	55.6	55.4	54.7	57.2
MRA	19.2	17.3^#^	23^#^	18.8	17.5^#^	22.3^#^
eGFR (Mean [SD])*	61.5(25.9)	62(25.3)	60.4 (25)	63.6 (24.2)	64.2(24.2) ^#^	62.2 (24.0)
eGFR > = 60 ml/min/1.73 m^2^	47.7	48.6	45.9	49.1	50.1	46.9
eGFR < 60 ml/min/1.73 m^2^	47.8	46.5	50.3	40.7	39.3	44.4
Missing/unknown	4.9	4.8	3.8	10.1	10.7	8.8

*Within 1 year prior to index date; ^#^significant differences between pts with and or without recurrent HF

ACEi angiotensin-converting enzyme inhibitor, AF atrial fibrillation, ARB angiotensin receptor blocker; CRT cardiac resynchronization therapy, CV cardiovascular, eGFR estimated glomerular filtration rate, HF heart failure, MRA mineralocorticoid receptor antagonist T2DM type 2 diabetes mellitus

Like our previous study, data during the 1-year pre-index period (including the index date) was used to assess comorbidities and CRT device use, and concomitant medications were assessed during the 3-month pre-index period [13]. The concomitant medications of interest in this cohort were angiotensin-converting enzyme inhibitor (ACEi)/angiotensin receptor blocker (ARB), mineralocorticoid receptor antagonist (MRA), beta-blocker (BB) and angiotensin receptor neprilysin inhibitor (ARNI),. The baseline tables, do not have data on ARNI as it was not approved until after November 2015. It should be noted that ARNI could be present as a time-dependent variable during the follow-up analysis in patients who were followed beyond November 2015 and who may have received prescriptions for ARNI [13].

During the study follow-up, CV mortality (where CV disease was listed as the primary cause of death and where CV disease was listed as any cause of death) and all-cause mortality for the HF population with AF and T2DM was recorded.

- Mortality outcomes were evaluated from the date of admission for the index HFH.
- Mortality events were assessed by linking the patients to the ONS database and establishing a date of death in the ONS database.
- All-cause mortality included all death events regardless of cause of death
- CV mortality as the primary cause of death was assessed using ICD-10 codes (i.e. Chapter I00-99) in the field with primary cause of death.
- CV mortality as any cause of death was assessed using ICD-10 codes (i.e. Chapter I00-99) in the field with primary cause of death and in any of the 15 causes of death fields as per the patient’s ONS file.

### Data analysis

Data analysis was on similar lines to our previous publication from the CPRD database [13]. In this study, both the demographic and clinical characteristics at baseline, were summarized using descriptive analyses. Imputation was not performed for the missing data. Either mean (standard deviation [SD]) or median (interquartile range [IQR]) were used to summarize continuous variables, and frequency and percentage were used to summarize categorical variables. Categorical variables were compared using the chi-square test and the Kolmogorov–Smirnov test was used to check the skewness of data. Continuous variables were compared using the unequal variance 2-sample t-test and Mann–Whitney U test was used for skewed datawas used [13]. An extended Cox regression model was used for reporting adjusted relative CV mortality as primary cause mortality rates and all -cause mortality rates for time-dependent recurrent HFH. The analysis included adjusted variables based on clinical importance or significance at baseline and if the missing values were below 30% [13].

## Results

### Baseline patient demographics and characteristics

A total of 116,262 patients with HF from the primary care database were considered for the study between January 01, 2006, and December 31, 2017. After applying the eligibility criteria, a total of 2344 patients with HF having T2DM and 4585 patients with HF having AF were included, contributing 4268.5 and 7845.6 patient-years, respectively. The baseline characteristics of patients with HF having T2DM and AF with and without recurrent HFH is shown in Table 1. Overall, 32.9% (n = 771) of T2DM patients and 28.2% (n = 1291) of AF patients had at least 1 recurrent HFH. The included patients with HF having T2DM and AF were relatively elderly, with a mean age of 76.4 and 79.6 years at index and were predominantly male (> 50%). The mean BMI was 30.9 in patients with HF having T2DM and 28.5 in patients with HF having AF. Overall, hypertension was the most common comorbid condition in both the cohorts: 76.5% in HF with T2DM and 67% in HF with AF patients, respectively. In both the cohorts, a higher proportion of patients with at least 1 recurrent HFH were more likely to have comorbidities and concomitant medications usage compared with patients without recurrent HFH.

### Hospitalizations and mortality rates

#### Patients with HF and T2DM

**Fig. 2:**
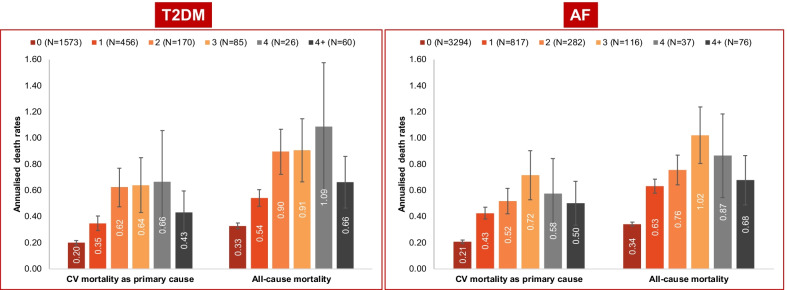
Annualized mortality rates according to the number of recurrent HF hospitalizations in T2DM and AF. AF, atrial fibrillation; CV, cardiovascular; HF, heart failure; T2DM, type 2 diabetes mellitus

The mean (SD) number of all-cause hospitalizations in patients with HF having T2DM and having ≥ 1 recurrent HFH was 10.8 (39.6), which was significantly higher than in patients without recurrent HFH: 4.5 (17.8). Overall, 53.8% of patients with HF having T2DM died from any cause (Table 2). Nearly 50% of deaths in the total cohort were attributed to CV mortality as any cause (where CV disease was listed as any cause of death) and more than one-third of deaths in the total cohort were attributed to CV mortality as the primary cause. Across the groups of patients with recurrent HFH, 48.7%–65.4% of the total deaths had CV disease listed as any cause (where CV disease was listed as any cause of death) and 34.9%–43.3% of total deaths had CV disease listed as the primary cause. The proportion of patients with all-cause mortality and mortality due to CV as any cause (where CV disease was listed as any cause of death) increased with increasing number of recurrent HFHs (up to 4 and decreased for ≥ 4 recurrent HFHs). The proportion of patients with mortality due to CV as the primary cause continued to increase with increasing number of recurrent HFHs (≥ 4 recurrent HFHs, Table 2). The annualized mortality rates in all-cause mortality and CV mortality as primary cause increased with each additional recurrent HFH until the fourth recurrent HF hospitalization and decreased thereafter (Fig. 2). Among the patients with ≥ 4 recurrent HFHs (n = 60), 66.7% (n = 40) of patients died by the end of the study, of which CV mortality as primary cause contributed to 65% (n = 26) of the total deaths. The mean time to all-cause mortality and CV mortality (where CV disease was listed as primary cause and any cause of death) in patients with HF having T2DM increased in up to 4 recurrent HFHs and decreased thereafter. The overall mean length of hospitalization leading to all-cause death in patients with HF having T2DM was 16.6 days. Similarly, overall mean length of hospitalization leading to CV mortality as primary cause and CV as any cause (where CV disease was listed as any cause of death) was 16.7 days.

**Table 2:** Proportion of deaths across study groups

	Death events	0 recurrent HFH	1 recurrent HFHs	2 recurrent HFHs	3 recurrent HFHs	4 recurrent HFHs	≥ 4 recurrent HFHs	Overall
T2DM	n	1573	456	170	85	26	60	2344
	All cause	824 (52.4)	248 (54.4)	99 (58.2)	51 (60.0)	18 (69.2)	40 (66.7)	1262 (53.8)
	CV (as any cause), n (%)	725 (46.1)	222 (48.7)	94 (55.3)	48 (56.5)	17 (65.4)	37 (61.7)	1126 (48.0)
	CV (as primary cause), n (%)	501 (31.9)	159 (34.9)	69 (40.6)	36 (42.4)	11 (42.3)	26 (43.3)	791 (33.8)
AF	n	3294	817	282	116	37	76	4585
	All cause	1755 (53.3)	483 (59.1)	159 (56.4)	80 (69.0)	27 (73.0)	46 (60.5)	2523 (55.0)
	CV (as any cause), n (%)	1535 (46.6)	444 (54.4)	154 (54.6)	75 (64.7)	26 (70.3)	43 (56.6)	2251 (49.1)
	CV (as primary cause), n (%)	1059 (32.2)	325 (39.8)	109 (38.7)	56 (48.3)	18 (48.7)	34 (44.7)	1583 (34.5)

AF atrial fibrillation, CV cardiovascular, HFH heart failure hospitalization, T2DM type 2 diabetes mellitus

#### Patients with HF and AF

The mean (SD) number of all-cause hospitalizations in patients with HF having AF with ≥ 1 recurrent HFH was 7 (14.6), which was significantly higher than in patients without recurrent HFH: 3.4 (9.4).

Overall, 55% of patients with HF having AF died from any cause (Table 2). Nearly 50% of deaths in the total cohort were attributed to CV mortality as any cause (where CV disease was listed as any cause of death) and more than one-third of deaths in the total cohort were attributed to CV mortality as the primary cause. Across the groups of patients with recurrent HFH, 54.4%–70.3% of the total cohort had CV disease as any cause of death (where CV disease was listed as any cause of death) and 39.8%–48.7% of total cohort had CV disease listed as the primary cause of death. The proportion of patients with all-cause mortality and mortality due to CV as any cause (where CV disease was listed as any cause of death)increased with an increase in up to 4 recurrent HFHs and decreased thereafter (Table 2). The annualized mortality rates in all-cause mortality and CV mortality as the primary cause increased with up to 3 recurrent HFHs, and decreased thereafter (Fig. 2). Among patients with ≥ 4 recurrent HFHs (n = 76), 60.5% (n = 46) of patients died by the end of the study, of which CV mortality as primary causecontributed to 73.9% (n = 34) of the total deaths. The mean time to all-cause mortality and CV mortality (both primary and all cause) in patients with HF having AF increased with an increase in up to 3 recurrent HFHs then showed a slight dip at the fourth recurrent HFH and increased thereafter. The overall mean length of hospitalization in patients with HF having AF was similar for all-cause mortality and CV mortality (where CV disease was listed as any cause of death) ( 17 days) and was shorter in patients having CV mortality as the primary cause (16.5 days).

### Association between recurrent HFH and CV mortality as primary cause and all-cause mortality

Recurrent HFH was associated with a statistically significant increase in all-cause mortality and CV mortality as primary cause for patients with HF having T2DM and patients with HF having AF. The risk of CV mortality as primary cause was 5.39 times higher for HF patients having T2DM with 3 recurrent HFHs versus no recurrent HFH. Similarly, the risk of all-cause mortality was 3.19 times higher for HF patients having T2DM with 3 recurrent HFHs versus no recurrent HFH. For HF patients having AF, the risk of CV mortality as primary cause was 5.98 times higher with 3 recurrent HFHs versus HF patients with no recurrent HFH. The risk of all-cause mortality was 4.31 times more for HF patients having AF with 3 recurrent HFHs versus no recurrent HFH.

**Fig. 3:**
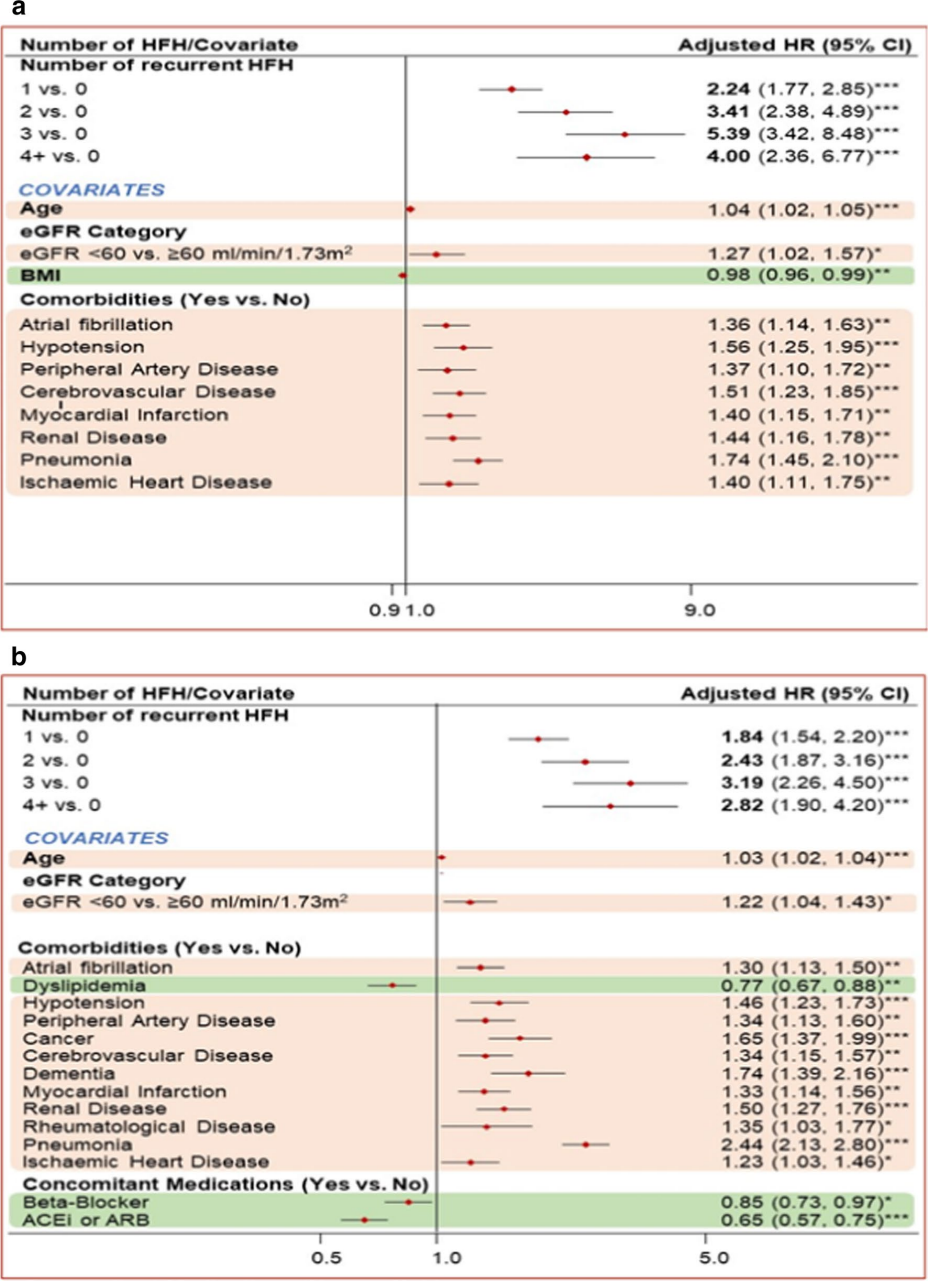
Impact of covariates on mortality in patients with HF and T2DM. **a** Impact of covariates on CV mortality as primary cause. **b** Impact of covariates on all-cause mortality. ***p < 0.001; **p < 0.01; *p < 0.05; ACEi, angiotensin-converting enzyme inhibitor; ARB, angiotensin receptor blocker; BMI, body mass index; CI, confidence interval; CV, cardiovascular; eGFR, estimated glomerular filtration rate; HF, heart failure; HFH, heart failure hospitalization; HR, hazard ratio; T2DM, type 2 diabetes mellitus

**Fig. 4:**
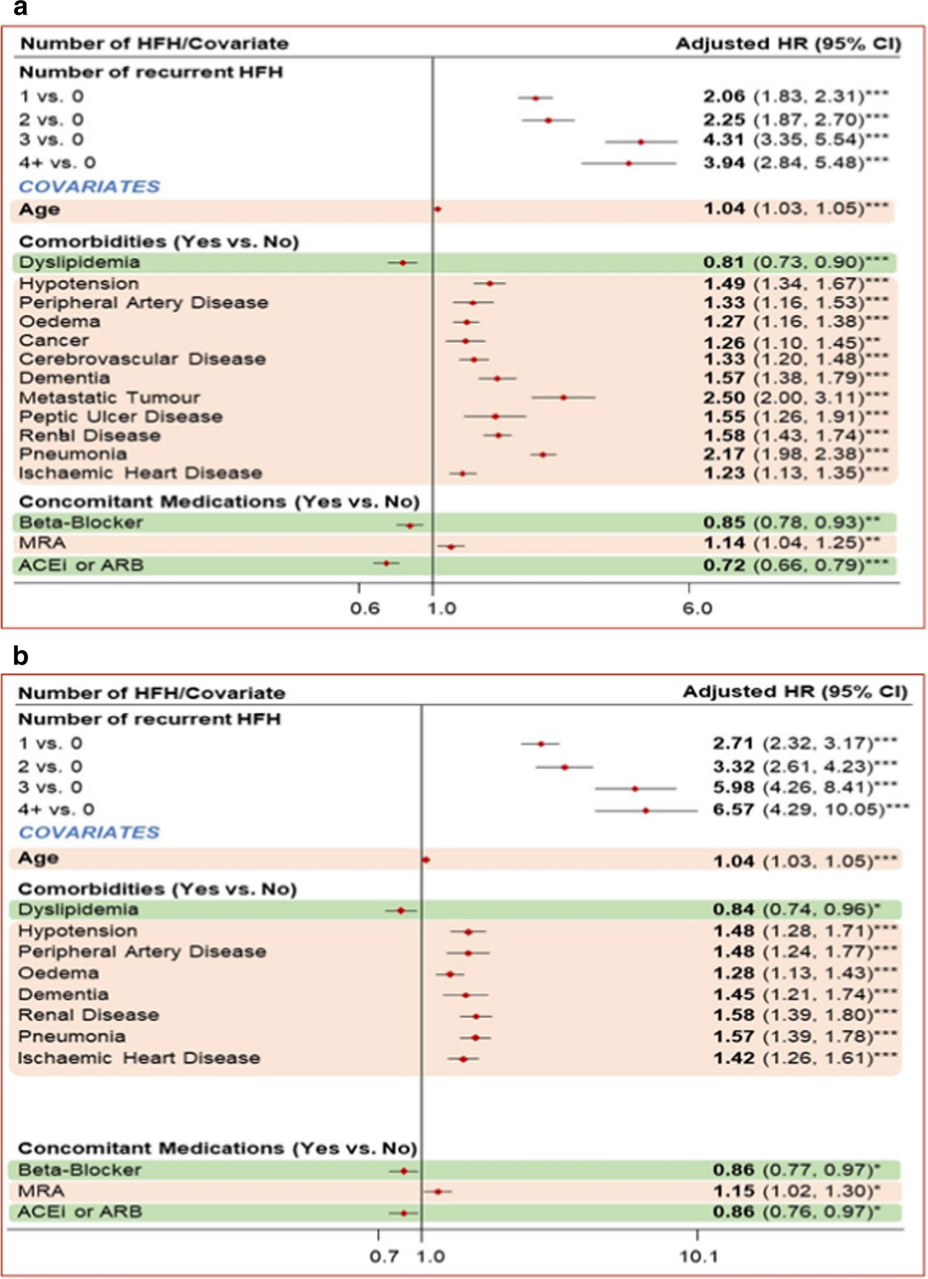
Impact of covariates on mortality in patients with HF and AF. **a** Impact of covariates on CV mortality as primary cause. **b** Impact of covariates on all-cause mortality. ***p < 0.001; **p < 0.01; *p < 0.05; ACEi, angiotensin-converting enzyme inhibitor; AF, atrial fibrillation; ARB, angiotensin receptor blocker; CI, confidence interval; CV, cardiovascular; HF, heart failure; HFH, heart failure hospitalization; HR, hazard ratio; MRA, mineralocorticoid receptor antagonist

An increase in age was associated with a significant increase in the risk of mortality (Figs. 3a, b, 4a, b).

- Patients with HF and T2DM; CV mortality as primary cause (hazard ratio [HR] 1.04; *p* < 0.001) and all-cause mortality (HR 1.03; *p* < 0.001)
- Patients with HF and AF; CV mortality as primary cause and all-cause mortality (HR 1.04; *p* < 0.00, for both)

After adjusting for various factors in the model, it was seen that in patients with HF and with T2DM and AF, various comorbid conditions contributed to an increased risk of mortality (Figs. 3a, b, 4a, b).

- For patients with HF and T2DM, pneumonia was associated with the largest increase in hazard for CV mortality as primary cause (HR 1.74; *p* < 0.001) and all cause (HR 2.44; *p* < 0.001)
- For patients with HF and AF, having a comorbid renal disease was the highest risk factor for CV mortality as the primary cause (HR 1.58; *p* < 0.001) and the presence of a metastatic tumor was the main contributor to all-cause mortality (HR 2.50; *p* < 0.001)

Additionally, while the use of ACEi/ARB and BB had a protective effect on all-cause mortality in patients with HF and with T2DM and AF, the use of MRA was associated with an increased risk of mortality in patients with HF and AF; for CV mortality as the primary cause (HR 1.15; *p* < 0.05) and all-cause mortality (HR 1.14; *p* < 0.01).

## Discussion

Results from previous publications involving CPRD records by Lahoz et al. have reported that the risk of mortality increases significantly with increasing number of recurrent HFHs in overall HF patients. To the best of our knowledge, this is the first study to quantify the magnitude of risk between recurrent HFH and mortality in patients with HF with comorbidities, such as T2DM and AF, from a real-world setting in the UK. This study highlights that recurrent HFHs are associated with an increased risk of CV and all-cause mortality.. The study population was taken from the CPRD, which has over 10 million active patient records drawn from approximately 1100 primary care practices across the UK, and thus can be considered representative of the UK population.

The analysis of this study showed that in HF patients living T2DM, the adjusted risk of CV mortality as primary causewasmore than twice in patients with 1 recurrent HFH and 5.39 times more for patients with 3 recurrent HFHs versus those with no recurrent HFH Similarly, the adjusted risk of all-cause mortality increased by 1.84 times in patients with 1 recurrent HFH and 3.19times in patients with 3 recurrent HFHs, compared with that in patients with no recurrent HFH. On similar lines, patients with HF and AF were at a 2.71 and 5.98 times at increased risk (adjusted) for CV mortality (as primary cause) if they had 1 and 3 recurrent HFHs, compared with no recurrent HFH. Likewise, this cohort had a two- and four-fold increased risk of all-cause mortality with 1 and 3 recurrent HFHs, compared with no recurrent HFH. To better understand the absolute increase in the risk of CV mortality and all-cause mortality, the baseline risk for these mortalities in the HF patient groups has to be considered in addition to the identified relative increase in risk for these mortalities.

The included patients were elderly (mean age of > 75 years), which is representative of the fact that HF is more common in the elderly and the occurrence of HF in this population is expected to increase in the future [4–6, 17]. Moreover, earlier studies evaluating the relationship of recurrent HFH with mortality or re-admissions following incident HFH too had patients with similar age characteristics [18, 19]. In addition to T2DM and AF, the relatively high age of the patients could be one of the contributing factors for them having comorbidities, recurrence of HF, and a high mean length of stay in the hospital of (more than 12 days), which results in a burden to the health care system.

A review by Correale et al. had previously discussed the impact of CV comorbidities such as hypertension, AF, coronary artery disease and non-CV comorbidities such as diabetes mellitus, cancer, chronic kidney disease etc. on chronic HF patients. According to this review there is a high prevalence of comorbidities in HF patients [20]. As comorbidities can negatively impact HF outcomes, managing comorbidities are equally important as managing HF.This study data too suggests that patients with HF having other comorbid conditions such as atrial fibrillation, hypotension, peripheral atrial disease, cerebrovascular disease, myocardial infraction, renal disease, pneumonia, ischemic heart disease, dementia etc.in addition to T2DM and AF were at a higher risk of both CV mortality (as primary cause) and all-cause mortality compared with patients not having comorbidities, highlighting the need to manage these comorbid conditions. The study shows that a higher proportion of patients with recurrent HFH in both cohorts had comorbidities and were using CV medications compared with patients without recurrent HFH, which may a reflection of disease severity in these patients. Furthermore, the mean number of all-cause hospitalizations in patients with HF having T2DM and AF with ≥ 1 recurrent HFH (10.8 and 7) were higher than those without recurrent HFH (4.5 and 3.4). These findings imply that patients with more comorbidities experience more hospitalizations in general as well as more recurrent hospitalizations due to HF specifically. Moreover, patients frequently hospitalized for HF are more prone to worsening of their general condition and this could also explain the higher mortality risk they have compared with patients without recurrent HFH.

A significant increase was noted in the annualized mortality rates for up to 4 recurrent HFHs in T2DM and for up to 3 recurrent HFHs in AF cohorts. The group with ≥ 4 recurrent HFHs consists of patients who had 4, 5, and 6 (up to 12) recurrent HFHs grouped together to ensure an adequate sample size.

The study shows the importance of recurrent HFH in relation to increasing the risk of mortality. Consequently, disease management for HF should also focus on reducing re-hospitalizations and might indirectly support improvement of the overall prognosis for the patients. Additionally, the various treatments in this study are likely a reflection of the different patient profiles. The use of MRAs was significantly associated with an increased risk of mortality in patients with HF and with AF. Though there was a higher absolute number of fatal events in patients with HF and with T2DM using MRAs, it was not significantly associated. The high mortality in MRA patients could be because the use of MRA can increase hyperkalemia and subsequent risk of arrhythmias leading to mortality (reference). Compared with this, BB and ACEi/ARB use was associated with a decreased risk of mortality.

## Limitations

Patients with a 4-year clean period are included, which is reasonable as the study identified the actual first HFH. HFHs prior to this clean period would not have been considered. There could be inherent limitations associated with the retrospective nature of the study such as lack of data to measure certain key variables of interest. The design could have resulted in certain bias in the form of selection bias or information bias that were beyond control. However, given the quality and breath of the CPRD, these should be minimal, but cannot be excluded., including missing data. Additionally, this study only included patients with at least 1 HFH, so potentially less severe patients not experiencing an HFH and severe patients who died before the first hospitalization were not covered by this study. As this study used patient identifiers from the CPRD database to link to the HES database, there is a risk of missing out on some severe patients with HF in the inpatient setting and where diagnosis was not recorded in the CPRD General practicioners (GPs). Information on HF severity (New York Heart Association classification) in both T2DM and AF patients was not available, so a relationship of recurrent HFH with mortality after adjustment for functional status could not be evaluated. Medication use might have been overestimated as the prescription of the drug was used as a proxy for use. Another limitation of this study is that the impact of all the approved medications in HF could not be adequately assessed because, interventions like ARNI was approved towards the end of the study period (after November 2015) and SGLT2 inhibitors were not approved during the study period. Moreover, many of the new treatments are indicated for subpopulations of HF. There is a need to profile the impact of these newly introduced medicines on their target population in an additional study, once sufficient follow-up time is available to capture the occurrence of hospitalization events Furthermore, variations in results due to the type of HF could not be determined as < 3% of the AF and T2DM HF cohorts had data on left ventricular ejection fraction. Due to this, the study did not differentiate between HF with reduced, preserved or midrange ejection fractions. Differences in these groups would be of interest, however, this was not possible with the available data and should be further assessed in the future.

## Conclusions

Recurrent HFHs are strongly associated with CV mortality and all-cause mortality as the risk of CV mortality and all-cause mortality increases progressively with each recurrent HFH in patients with HF and TD2M or HF and AF.

Mortality was even high among all patients, including in those without recurrent HFHs. The number of hospitalizations is associated with prognosis and highlights the need for treatment and disease management, which will improve the course of the disease and help patients stay out of hospital.

## Supplementary Information

**Additional file 1**. Primary care coding schemes for identifying HF diagnoses and HFHs.

## Data Availability

The datasets generated and analyzed during the current study are available from the corresponding author on reasonable request.

## Abbreviations

ACEi: Angiotensin-converting enzyme inhibitor
AF: Atrial fibrillation
ARB: Angiotensin receptor blocker
ARNI: Angiotensin receptor neprilysin inhibitor
BB: Beta-blocker
BMI: Body mass index
CPRD: Clinical Practice Research Datalink
CRT: Cardiac resynchronization therapy
CV: Cardiovascular
eGFR: Estimated glomerular filtration rate
EU: European Union
HES: Hospital Episode Statistics
HF: Heart failure
HFH: HF hospitalizations
HR: Hazard ratio
ICD-10: International Classification of Diseases, 10th Revision
IMD: Index of Multiple Deprivation
IQR: Interquartile range
ISAC: Independent Scientific Advisory Committee
MRA: Mineralocorticoid receptor antagonist
NHS: National Health Service
NYHA: New York Heart Association
ONS: Office of National Statistics
SD: Standard deviation
T2DM: Type 2 diabetes mellitus
UK: United Kingdom
US: United States
