# Supplementary material for: Recurrent heart failure hospitalizations increase the risk of mortality in heart failure patients with atrial fibrillation and type 2 diabetes mellitus in the United Kingdom: a retrospective analysis of Clinical Practice Research Datalink database
Source: BMC Cardiovasc Disord. 2022 May 21;22:234. doi: 10.1186/s12872-022-02665-y (PMC9124377; doi:10.1186/s12872-022-02665-y)
Supplement: Supplementary file 1 — Additional file 1. Primary care coding schemes for identifying HF diagnoses and HFHs. [file 12872_2022_2665_MOESM1_ESM.docx]

**Table 1: Primary care coding schemes for identifying HF diagnoses and HFHs**

| **local dictionary** | **local code** | **local description** | **medcode** |
| --- | --- | --- | --- |
| Read | G1yz100 | Rheumatic left ventricular failure | 22262 |
| Read | G232.00 | Hypertensive heart and renal disease with (congestive) heart failure | 21837 |
| Read | G58..00 | Heart failure | 2062 |
| Read | G58..11 | Cardiac failure | 1223 |
| Read | G580.00 | Congestive heart failure | 398 |
| Read | G580.11 | Congestive cardiac failure | 2906 |
| Read | G580.14 | Biventricular failure | 9524 |
| Read | G580000 | Acute congestive heart failure | 23707 |
| Read | G580100 | Chronic congestive heart failure | 32671 |
| Read | G580200 | Decompensated cardiac failure | 27884 |
| Read | G580300 | Compensated cardiac failure | 11424 |
| Read | G580400 | Congestive heart failure due to valvular disease | 94870 |
| Read | G581.00 | Left ventricular failure | 884 |
| Read | G581.11 | Asthma - cardiac | 23481 |
| Read | G581.13 | Impaired left ventricular function | 5942 |
| Read | G581000 | Acute left ventricular failure | 5255 |
| Read | G582.00 | Acute heart failure | 27964 |
| Read | G58z.00 | Heart failure NOS | 4024 |
| Read | G58z.11 | Weak heart | 12590 |
| Read | G58z.12 | Cardiac failure NOS | 17278 |
| Read | 1O1..00 | Heart failure confirmed | 9913 |
| Read | 388D.00 | New York Heart Association classification of heart failure symptoms | 46672 |
| Read | 661M500 | Heart failure self-management plan agreed | 106198 |
| Read | 662p.00 | Heart failure 6 month review | 83502 |
| Read | 662T.00 | Congestive heart failure monitoring | 12366 |
| Read | 662W.00 | Heart failure annual review | 30779 |
| Read | 8CMK.00 | Has heart failure management plan | 103732 |
| Read | 8H2S.00 | Admit heart failure emergency | 32898 |
| Read | 8Hg8.00 | Discharge from practice nurse heart failure clinic | 91288 |
| Read | 8HHz.00 | Referral to heart failure exercise programme | 70619 |
| Read | 8Hk0.00 | Referred to heart failure education group | 71235 |
| Read | 8HTL.00 | Referral to heart failure clinic | 48897 |
| Read | 9hH..00 | Exception reporting: heart failure quality indicators | 90935 |
| Read | 9hH0.00 | Excepted from heart failure quality indicators: Patient unsuitable | 30749 |
| Read | 9hH1.00 | Excepted from heart failure quality indicators: Informed dissent | 64062 |
| Read | 9N0k.00 | Seen in heart failure clinic | 12627 |
| Read | 9N2p.00 | Seen by community heart failure nurse | 19002 |
| Read | 9N4s.00 | Did not attend practice nurse heart failure clinic | 95021 |
| Read | 9N4w.00 | Did not attend heart failure clinic | 83481 |
| Read | 9N6T.00 | Referred by heart failure nurse specialist | 69062 |
| Read | 9Or0.00 | Heart failure review completed | 19380 |
| Read | 9Or1.00 | Heart failure monitoring telephone invite | 90193 |
| Read | 9Or2.00 | Heart failure monitoring verbal invite | 90192 |
| Read | 9Or3.00 | Heart failure monitoring first letter | 72965 |
| Read | 9Or4.00 | Heart failure monitoring second letter | 72386 |
| Read | 9Or5.00 | Heart failure monitoring third letter | 89650 |
| Read | ZRad.00 | New York Heart Assoc classification heart failure symptoms | 26242 |
| Read | G5y4z00 | Post cardiac operation heart failure NOS | 96799 |
| Read | SP11111 | Heart failure as a complication of care | 66306 |
| Read | 679W100 | Education about deteriorating heart failure | 105002 |
| Read | 67D4.00 | Heart failure information given to patient | 60099 |
| Read | 8HgD.00 | Discharge from heart failure nurse service | 102585 |
| Read | 8HHb.00 | Referral to heart failure nurse | 26115 |
| Read | 8HTL000 | Referral to rapid access heart failure clinic | 106680 |
| Read | 14A6.00 | H/O: heart failure | 15058 |
| Read | 14AM.00 | H/O: Heart failure in last year | 46912 |
| Read | G55..00 | Cardiomyopathy | 3204 |
| Read | 33BA.00 | Impaired left ventricular function | 7251 |
| Read | G551.00 | Hypertrophic obstructive cardiomyopathy | 8010 |
| Read | G554400 | Primary dilated cardiomyopathy | 7535 |
| Read | G343.00 | Ischaemic cardiomyopathy | 7320 |
| Read | 662h.00 | New York Heart Association classification - class III | 19066 |
| Read | G554300 | Hypertrophic non-obstructive cardiomyopathy | 3499 |
| Read | G55z.00 | Cardiomyopathy NOS | 22993 |
| Read | G5yyD00 | Left ventricular cardiac dysfunction | 107397 |
| Read | 662f.00 | New York Heart Association classification - class I | 18853 |
| Read | G583.00 | Heart failure with normal ejection fraction | 101138 |
| Read | G555.00 | Alcoholic cardiomyopathy | 4915 |
| Read | G55y.11 | Secondary dilated cardiomyopathy | 9402 |
| Read | G554200 | Familial cardiomyopathy | 21852 |
| Read | G583.12 | Heart failure with preserved ejection fraction | 106897 |
| Read | G559.00 | Arrhythmogenic right ventricular cardiomyopathy | 97780 |
| Read | G583.11 | HFNEF - heart failure with normal ejection fraction | 101137 |
| Read | 8CMW800 | Heart failure clinical pathway | 106008 |
| Read | G558100 | Cardiomyopathy in myotonic dystrophy | 27683 |
| Read | Gyu5M00 | [X]Other hypertrophic cardiomyopathy | 70648 |
| Read | 662i.00 | New York Heart Association classification - class IV | 51214 |
| Read | G21z100 | Hypertensive heart disease NOS with congestive cardiac failure | 62718 |
| Read | G211100 | Benign hypertensive heart disease with congestive cardiac failure | 52127 |
| Read | 8CeC.00 | Preferred place of care for next exacerbation of heart failure | 105542 |
| Read | 1736.00 | Paroxysmal nocturnal dyspnoea | 6434 |
| Read | 1J60.00 | Suspected heart failure | 21235 |
| Read | 23E1.00 | O/E - pulmonary oedema | 5155 |
| Read | 679X.00 | Heart failure education | 95835 |
| Read | 8HBE.00 | Heart failure follow-up | 17851 |
| Read | 9On0.00 | Left ventricular dysfunction monitoring first letter | 60710 |
| Read | 9On1.00 | Left ventricular dysfunction monitoring second letter | 60721 |
| Read | 9On2.00 | Left ventricular dysfunction monitoring third letter | 72341 |
| Read | 9On3.00 | Left ventricular dysfunction monitoring verbal invite | 92305 |
| Read | 9On4.00 | Left ventricular dysfunction monitoring telephone invite | 96484 |
| Read | 9h1..00 | Exception reporting: LVD quality indicators | 34213 |
| Read | 9h11.00 | Excepted from LVD quality indicators: Patient unsuitable | 11613 |
| Read | 9h12.00 | Excepted from LVD quality indicators: Informed dissent | 28649 |
| Read | G581.12 | Pulmonary oedema - acute | 43618 |
| Read | H54..00 | Pulmonary congestion and hypostasis | 30214 |
| Read | H541.00 | Pulmonary congestion | 1585 |
| Read | H541000 | Chronic pulmonary oedema | 26082 |
| Read | H541z00 | Pulmonary oedema NOS | 7321 |
| Read | H54z.00 | Pulmonary congestion and hypostasis NOS | 61229 |
| Read | H584.00 | Acute pulmonary oedema unspecified | 558 |
| Read | H584z00 | Acute pulmonary oedema NOS | 5293 |
| Read | G210.00 | Malignant hypertensive heart disease | 50157 |
| Read | G210000 | Malignant hypertensive heart disease without congestive cardiac failure | 95334 |
| Read | G210100 | Malignant hypertensive heart disease with congestive cardiac failure | 72668 |
| Read | G230.00 | Malignant hypertensive heart and renal disease | 67232 |
| Read | G234.00 | Hypertensive heart and renal disease with both (congestive) heart failure and renal failure | 57987 |
| Read | G400.00 | Acute cor pulmonale | 8464 |
| Read | G41z.11 | Chronic cor pulmonale | 5695 |
| Read | G554000 | Congestive cardiomyopathy | 5141 |
| Read | G554011 | Congestive obstructive cardiomyopathy | 68766 |
| Read | G580.12 | Right heart failure | 10079 |
| Read | G580.13 | Right ventricular failure | 10154 |
| Read | R2y1000 | [D]Cardiorespiratory failure | 20324 |
| Read | 662g.00 | New York Heart Association classification - class II | 13189 |
| ICD10 | I11.0 | Hypertensive heart disease with (congestive) heart failure | NULL |
| ICD10 | I13.0 | Hypertensive heart and renal disease with (congestive) heart failure | NULL |
| ICD10 | I13.2 | Hypertensive heart and renal disease with both (congestive) heart failure and renal failure | NULL |
| ICD10 | I42.0 | Dilated cardiomyopathy | NULL |
| ICD10 | I42.1 | Obstructive hypertrophic cardiomyopathy | NULL |
| ICD10 | I42.2 | Other hypertrophic cardiomyopathy | NULL |
| ICD10 | I42.9 | Cardiomyopathy, unspecified | NULL |
| ICD10 | I50 | Heart failure | NULL |
| ICD10 | I50.0 | Congestive heart failure | NULL |
| ICD10 | I50.1 | Left ventricular failure | NULL |
| ICD10 | I50.9 | Heart failure, unspecified | NULL |
